# Reply to Graña et al. Comment on “Uzun Ozsahin et al. COVID-19 Prediction Using Black-Box Based Pearson Correlation Approach. *Diagnostics* 2023, *13*, 1264”

**DOI:** 10.3390/diagnostics14222529

**Published:** 2024-11-12

**Authors:** Dilber Uzun Ozsahin, Efe Precious Onakpojeruo, Basil Bartholomew Duwa, Abdullahi Garba Usman, Sani Isah Abba, Berna Uzun

**Affiliations:** 1 Department of Medical Diagnostic Imaging, College of Health Science, University of Sharjah, Sharjah 27272, United Arab Emirates; 2 Research Institute for Medical and Health Sciences, University of Sharjah, Sharjah 27272, United Arab Emirates; 3 Operational Research Centre in Healthcare, Near East University, TRNC Mersin 10, Nicosia 99138, Turkey; efeprecious.onakpojeruo@neu.edu.tr (E.P.O.); basil.barthduwa@neu.edu.tr (B.B.D.); abdullahigarba.usman@neu.edu.tr (A.G.U.); berna.uzun@neu.edu.tr (B.U.); 4 Department of Biomedical Engineering, Near East University, TRNC Mersin 10, Nicosia 99138, Turkey; 5 Department of Analytical Chemistry, Faculty of Pharmacy, Near East University, TRNC Mersin 10, Nicosia 99138, Turkey; 6 Department of Civil Engineering, Prince Mohammad Bin Fahd University, Al Khobar 31952, Saudi Arabia; sabba@pmu.edu.sa; 7 Department of Mathematics, Near East University, TRNC Mersin 10, Nicosia 99138, Turkey

Dr. Graña et al. have made some comments relating to the standard of the published article titled “COVID-19 Prediction Using Black-Box Based Pearson Correlation Approach” [1]. We sincerely appreciate the constructive criticism and acknowledge the need for clarity in the Methodology description and data handling in the publication. Moreover, the critics should equally understand that the manuscript has passed through a blind peer-review process, which involves certain critiques by the reviewers and answers from the authors.

## 1. Title

The critic has raised concerns regarding the use of the term “Black-Box Based Pearson Correlation Approach” in the title. Indeed, there is no confusion regarding the use of the two terms as the critical review claimed, owing to the fact that our study incorporates black box ML techniques in the form of ANN and MLR coupled with ideas derived from Pearson correlation, which measures the linear relationship between two variables, as demonstrated in Figure 6. Figure 6 demonstrates the linear relationship between vaccination influence and COVID-19 mortality cases in Israel.

## 2. Methodology

### 2.1. Research Questions

The critic expresses reservations over the level of addressing research questions and drawing comparisons between Israel and Greece. For instance, research question (i) compares the weekly COVID-19 cases between Israel and Greece; Figures 2 and 4 depict the comparative graphical representation in the form of a visualization that compares both weekly COVID-19 cases between Greece and Israel, respectively.

The primary objective of this study was to conduct a comparative analysis of the weekly COVID-19 case data from Israel and Greece. The second objective of the study was to analyze the disparities in monthly COVID-19 mortality cases between Israel and Greece. Thirdly, the objective was to ascertain the association between the vaccination rate in Israel and the incidence of COVID-19-related fatalities and then provide the obtained results. Finally, to predict the occurrence of COVID-19 cases in Israel. The research questions exhibit a clear emphasis on Israel. The advantage of performing these activities is that several mitigations can be put in place, lowering the risk that the infection would propagate. A correlation analysis was conducted in the study, along with the implementation of two distinct AI models: ANN and MLR. The objective of both models was to predict the number of COVID-19 cases in Israel, utilizing correlated data as input factors. In order to evaluate the practical performance of each model, only datasets from Israel were used to train the model for the prediction. Furthermore, the data for Greece was added to statistically compare its weekly COVID-19 cases with that of Israel since the two countries had a comparatively similar number of cases reported at that time (see Figures 2–5). Moreover, we employed the use of four evaluation metrics, including the determination coefficient (R^2^), mean squared error (MSE), root mean squared error (RMSE), and correlation coefficient (R). Hence, a comparison was made during the study and reported in the descriptive analysis section of the paper.

### 2.2. Dataset and Data Pre-Processing

The critic raises questions pertaining to the provenance of the dataset, the characteristics of the variables, and the absence of details regarding the pre-processing of the data.

It was mentioned that the dataset was sourced from Kaggle. The data types and variables were described, and the data normalization was explained to meet a custom range. Data imputation and missing value handling were acknowledged but not detailed for brevity and relevancy. A comprehensive explanation of the data pre-processing procedures may be found in the main manuscript, in the section titled “Filtering and Pre-Processing the Data”. The complete dataset pertaining to COVID-19, specifically the coronavirus, can be accessed at the following URL: https://www.kaggle.com/datasets/kalilurrahman/covid19-coronavirus-dataset-by-owid accessed on 2 January 2023 [2].

The dataset, namely owid-covid-data.csv, was acquired on 1 June 2022 from the following source: https://www.kaggle.com/datasets/kalilurrahman/covid19-coronavirus-dataset-by-owid accessed on 2 January 2023. This comprehensive COVID-19 dataset comprises the COVID-19 data curated by Our World in Data—Statistics and Research, and the individuals responsible for the data include Hannah Ritchie, Esteban Ortiz-Ospina, Diana Beltekian, Edouard Mathieu, Joe Hasell, Bobbie Macdonald, Charlie Giattino, and Max Roser. The web development of the dataset was undertaken by BreckYunits, Ernst van Woerden, Daniel Gavrilov, Matthieu Bergel, Shahid Ahmad, and Jason Crawford.

## 3. Validation

The critics mention their confusion regarding the validation techniques. This was referenced in the study to illustrate the process of partitioning the dataset into separate training and testing sets. As described in Section 2.4 (Model Validation), the study utilizes four-fold cross-validation as the optimum method that provides the best performance results for the models by partitioning the data into a ratio of 70–30% for training and testing phases, respectively.

### 3.1. Experimental Design

The experimental strategy and methods are fully described in the study. In order to predict the number of COVID-19 weekly cases, the study employed the use of two machine learning techniques, MLR and ANN, to comparatively compare their performance in both the training and testing stages, respectively, using various metrics including RMSE, MSE, R, and R^2^.

The experimental design provided an equal opportunity to visualize and compare the weekly number of COVID-19 cases recorded in both Israel and Greece, as they had a comparatively similar number of cases.

Moreover, the experimental design equally demonstrates the validation process used. The simulation in the form of MATLAB 9.3 (R2019A) was described for the simulation process in Section 3.

The percentage of the results was equally demonstrated based on the percentages of the R and R^2^-values, not just the R-values. For instance, we indicate in the Results and Discussion sections that for R^2^ and R, the training results for ANN were 84% and 91%, respectively, while the results for MLR were 97% and 98%, respectively. Furthermore, for the testing phase results, R^2^ and R for ANN were 94% and 97%, and the results for MLR were 97% and 98%, respectively.

Furthermore, the current study equally compares the performance of our results with the latest studies reported in the recently published literature in the Results and Discussion sections. We present and organize the findings from our predictive comparison in the following way: regarding the prediction of COVID-19, MLR was superior to ANN.

Therefore, this result is similar to the findings of [6,7,23,24,33–35], which depict the good performance of MLR, though it is a linear model.

### 3.2. Results Reporting

Concerning comments on the reporting of the study’s results, we appreciate the criticism expressed in those comments.

However, the major motivation of the current work was to employ two different MLs in order to predict COVID-19 cases but not mortality cases, as indicated in the other sections of the manuscript. Therefore, there is no need to provide further statistical tests as the modeling step involves several iterations of the models to achieve the optimum and desired results [3,4]. Moreover, we employed both performance fitness (R^2^ and R) as well as error performance metrics in the form of RMSE and MSE to depict the deviations in the actual values with the simulation values.

Moreover, as demonstrated by various recent publications, visualizations are used to provide concise knowledge of the data used in any study, elucidate data anomalies, and model anomalies such as overfitting, underfitting, local minima, etc. Therefore, in the current study, various graphs were presented for the easy assimilation and understanding of the obtained results for various readers of the article, including non-experts [5,6].

Concerns are mentioned with regard to the interpretation of correlations. Instead of assuming a direct causal relationship, the separation between dependent and independent variables was made for demonstration and visualization purposes. Correlation coefficients were calculated to display connections in the data; however, causality was not meant to be inferred.

Regarding the research question (III) about the relationship between the number of Israelis who were vaccinated and the number of Israelis who died, this inspired the creation of Figure 6. The association between the two variables led the authors to conclude that vaccination rates did not affect mortality.

Moreover, the output charts demonstrated in Figures 7–10 are meant to give the reader a bird’s-eye view of the data and the relationships between the observed and predicted values. The goal was to show connections within the data between the simulated and observed values.

## 4. AI Writing

The critic raises concerns related to the use of AI-generated text in the publication. We appreciate the constructive critique and recognize the need to express clarity in that regard.

As of when this paper was written and submitted to the journal, there was no provision for an AI writing disclosure by the journal, nor was there any reliable means of checking for AI similarity; hence, the authors only utilized the use of ChatGPT for grammar checks and other proofreading purposes. Moreover, we believe the plagiarism score was exaggerated as we have checked the plagiarism using Turnitin, and the results appear to be only 23%.

## 5. Discussion and Conclusions

We value the critique’s comments on the Discussion and Conclusions. The published study has a section titled “Application of Results and Discussion”, descriptive analysis, and results from the models in addition to the Conclusion, where the results are discussed. Therefore, the Conclusion is not a bunch of uneducated tidbits, as the comment suggests, but for the sake of brevity and relevance.

## Data Availability

All materials used in this study can be found in [2].

## Abbreviations

These abbreviations were utilized in the current work:

ANN	artificial neural network
AI	artificial intelligence
ML	machine learning
MLR	multi-linear regression
R^2^	determination coefficient
MSE	mean squared error
RMSE	root mean squared error
R	correlation coefficient

## References

[1] Graña M., Badiola-Zabala G., Cano-Escalera G. (2024). Comment on Uzun Ozsahin et al. COVID-19 Prediction Using Black-Box Based Pearson Correlation Approach. *Diagnostics* 2023, *13*, 1264. Diagnostics.

[2] COVID-19 Dataset by Our World in Data. https://www.kaggle.com/datasets/bolkonsky/covid19.

[3] Palm C., Connolly C.E., Masser R., Padberg Sgier B., Karamitopoulou E., Simon Q., Bode B., Tinguely M. (2023). Determining HER2 Status by Artificial Intelligence: An Investigation of Primary, Metastatic, and HER2 Low Breast Tumors. Diagnostics.

[4] Al-Hejri A.M., Al-Tam R.M., Fazea M., Sable A.H., Lee S., Al-Antari M.A. (2023). ETECADx: Ensemble Self-Attention Transformer Encoder for Breast Cancer Diagnosis Using Full-Field Digital X-ray Breast Images. Diagnostics.

[5] Labinsky H., Ukalovic D., Hartmann F., Runft V., Wichmann A., Jakubcik J., Gambel K., Otani K., Morf H., Taubmann J. (2023). An AI-Powered Clinical Decision Support System to Predict Flares in Rheumatoid Arthritis: A Pilot Study. Diagnostics.

[6] Kerexeta J., Larburu N., Escolar V., Lozano-Bahamonde A., Macía I., Beristain Iraola A., Graña M. (2023). Prediction and Analysis of Heart Failure Decompensation Events Based on Telemonitored Data and Artificial Intelligence Methods. J. Cardiovasc. Dev. Dis..

